# Labor Analgesia reduces the risk of postpartum depression: A cohort study

**DOI:** 10.1515/tnsci-2020-0193

**Published:** 2021-10-25

**Authors:** Li Ren, Qibin Chen, Su Min, Fangliang Peng, Bin Wang, Jian Yu, Yuxi Zhang

**Affiliations:** Department of Anesthesiology, The First Affiliated Hospital of Chongqing Medical University, No. 1 Youyi Road, Yuzhong District, Chongqing, China; Department of Obstetrics, The First Affiliated Hospital of Chongqing Medical University, Chongqing, China

**Keywords:** postpartum depression, labor analgesia, cesarean section

## Abstract

**Background:**

Postpartum depression (PPD) is a frequent mental disorder after delivery. In China, most parturients give birth with the assistance of labor analgesia (LA) or by cesarean section (CS); however, it is still unclear whether these two approaches reveal different effects on PPD.

**Methods:**

One hundred and ninety-eight patients with single pregnancy at full term were allocated to receive either group LA or group CS. Maternal and neonatal variables in the perinatal period were recorded. Multivariate logistical regression analysis was conducted to evaluate the associated factors of PPD.

**Results:**

The incidence of PPD in group LA was lower than in group CS. Besides, eight factors were found to be potential predictors of PPD. Multivariate logistic model showed that LA was a protective factor against PPD. However, high family income and Edinburgh postnatal depression scale (EPDS) scores at 3 days postpartum were associated with an increased risk of PPD.

**Conclusion:**

LA could reduce the incidence of PPD in women with single pregnancy at full term. Family income and EPDS scores in the early postpartum period were also related with PPD. Large sample size studies are needed to verify the impact of LA on the psychological states of postpartum women.

## 1 Introduction

During pregnancy, women will experience substantial changes in reproductive system. In addition, women are also subject to adaptive psychological alterations during the perinatal period. It is during this period that they begin to move toward parenthood, understand the needs of newborns, and adapt new social and family roles and relationships, which makes them vulnerable to mental illness [1]. Among all the mental disorders related to such transformation, postpartum depression (PPD) is one of the most frequent related to childbearing, and the World Health Organization defined it as moderate to severe depressive episodes that typically begin within six weeks after delivery [2], while the time frame is shortened to four weeks after delivery according to the American Psychiatric Association [3]. A recent survey on 56 countries indicated the global pooled prevalence of PPD was 17.7%, ranging from 3% in Singapore to 38% in Chile [4]. In general, the prevalence of PPD is lower in developed countries compared to developing countries [5,6]. Mothers with PPD tend to demonstrate poor social and psychological functions, which could compromise parent-child attachment [7]. In addition, PPD would also bring negative impacts on infants and families, and previous studies have reported close connection of PPD in mother and paternal PPD [8]. Moreover, infants are more likely to be stunted and vulnerable to physical disorders if he/she is living with the mother with PPD [9,10].

Etiology of PPD is complex and remains poorly understood, which includes biological, genetics, and psychosocial factors. Recent studies have made great achievement in exploring the potential neurobiological and genetics underpinnings of PPD [11,12]. However, the need to identify women who are vulnerable to PPD is still great for early detection and prevention of the disorder. Several potential risk factors have been identified, including history of depression, smoking during pregnancy, antenatal depression and anxiety, previous psychiatric illness, poor marital relationship, stressful life events, negative attitude towards pregnancy, lack of social support, and perinatal complications [6,13,14,15]. However, the topic of whether the delivery mode affects the risk of PPD remains controversial. Eckerdal et al. found that the delivery mode has no direct impact on the risk of PPD [16], while Xu et al. reported that the incidence of PPD among women who gave birth by cesarean section (CS) was significantly higher than those who delivered through vaginal delivery [17].

Labor analgesia (LA) is a process in which psychotherapy or therapeutic agents are used to reduce the pain in delivery. Epidural LA provides excellent pain relief during vaginal delivery; as a result, it has become one of the most widely used pain control strategies for childbirth [18]. In China, after the professional evaluation of obstetricians and anesthesiologist, patients without contraindication actually are liberal to make their own decision on their delivery mode. Accordingly, CS and LA have become the predominant birth practices due to no pain during delivery in many medical centers. Previous studies suggested that the intensity of perinatal pain is related to postpartum mood disorder or depression [19,20]. Apparently, women who adopt LA or CS experience labor pain and acute postpartum pain diversely. However, little evidence has been proposed to distinguish these two practices in terms of their potential to trigger PPD.

The aim of this prospective cohort study was to investigate the effects of LA and CS on PPD using the Edinburgh postnatal depression scale (EPDS) scores at 6 weeks after giving birth. Moreover, we further explored the predicted factors of PPD during the perinatal period.

## 2 Materials and methods

The study was conducted in the First Affiliated Hospital of Chongqing Medical University between January 2019 and November 2019.

### 2.1 Subjects

The inclusion criteria were as follows: pregnant women aged 18–45 years with singleton fetus more than 37 weeks, who were admitted to receive epidural LA-assisted delivery or elective CS. The exclusion criteria included: (1) patients with definitive diagnosed mental illness (e.g., schizophrenia, depression, anxiety, paranoia, mania, post-traumatic disorder, and obsessive-compulsive disorder), (2) CS with general anesthesia, (3) contradiction for epidural anesthesia (including coagulation disorder, infection at the puncture site, and lumbar injury), (4) local anesthetics allergy, and (5) severe illness complicating pregnancy (including severe hypertension, diabetes, or other severe heart, hepatic, and renal disorders). Participants were also excluded if they were bleeding over 800 mL or had a cardiac arrest during birth. In addition, patients with incomplete epidural anesthesia who needed general anesthesia during delivery or those who turned to CS due to failure of natural childbirth were not included in this study to avoid the bias brought by accidental psychological fluctuations.

### 2.2 Groups

Upon hospital admission, all eligible parturients were informed about the study, and informed consent was obtained from each parturient. Information about the advantage and disadvantage of LA and CS were also provided to the participants. Given the professional suggestions from doctors, each parturient made her own decision to receive either group LA or group CS. Subjects in group LA received epidural LA in the delivery room before birth, while subjects in group CS received epidural anesthesia in the operation room before surgery.

### 2.3 Epidural LA

Each participant had their cervix checked by a midwife or an obstetrician every 4 h during the latent phase of the first stage. When the cervix dilated to a width of 2 cm or more, epidural LA was administered by a senior anesthesiologist (QB Chen). The parturient would be placed in a lateral position, and L2–L3 interspace was chosen for puncture and catheterization. An initial dose of 5 mL of 1% lidocaine was administrated. When anesthesia was in effect and no adverse event or anesthetic intoxication occurred, a patient-controlled epidural analgesia (PCEA) pump would be connected to the catheter. The formulation of PCEA included 150 mg ropivacaine and 0.25 mg fentanyl dissolved in saline to a solution of 150 mL. The patient received a loading dose (8 mL) of PCEA as the connection completed, followed by a solution infusion rate of 8 mL/h with a bolus of 8 mL and a lock time of 20 min. The anesthesiologist was responsible to solve any side effects related to epidural analgesia. Epidural LA would be canceled upon the birth of the fetus.

### 2.4 Epidural anesthesia for elective CS

Epidural anesthesia was conducted by a senior anesthesiologist (B Wang) for each parturient who needed an elective CS. First, vein puncture and catheterization were performed by a nurse after the patient was sent to the operation room. Similar to epidural LA, the parturient was placed in a lateral position, but L1–L2 interspace was chosen for puncture and catheterization instead of L2–L3. An initial dose of 3–5 mL of 2% lidocaine was administrated to test the anesthetic effect and potential adverse events. When the responses were positive, 10–15 mL of 2% lidocaine with 1:200,000 epinephrine was subsequently administered. If the anesthesia was insufficient, 5–7 mL of 1% ropivacaine was added via the epidural catheter. The CS was performed by a senior obstetrician (FL Peng) through a transverse lower uterine segment incision when the level of anesthesia was between T6 and S3. After the fetus was taken out, the uterine wound was closed by continuous absorbable suture, and oxytocin or ergometrine was used if necessary. The skin incision was closed with nonabsorbable stitches, which were removed on day 7–10 after the operation. All patients received patient-controlled intravenous analgesia (PCIA) at the end of surgery. The formulation of PCIA included 800 mg tramadol and 40 mg nefopam dissolved in saline to a solution of 80 mL, and the administration of PCIA adopted a loading dose of 5 mL, an infusion rate of 1 mL/h, a bolus dose of 5 mL, and a lock time of 20 min.

### 2.5 General data collection

The demographic characteristics of the participants, including age, body mass index (BMI), education background, and information on the present pregnancy (including gravidity and parity, perinatal classes about childbirth, present obstetrical and gynecological disease, and diseases complicating pregnancy) were collected with a designed information form after receiving the written consent from the subjects. In addition, the baseline of the numerical rating scale (NRS) scores was collected for each parturient. The baseline of pain was assessed by NRS before the administration of epidural LA in group LA and before epidural anesthesia in group CS. Besides, antenatal depression was evaluated by the EPDS, which is a widely used 10-item questionnaire to detect perinatal depression, and the maximum score of an EPDS questionnaire is 30 [21,22]. Recently, the EPDS has been shown to be an effective screening tool for antenatal depression, A score of 10 or 11 in EPDS is considered a cut-off point to identify antenatal depression [23,24]. In the present study, antenatal depression was defined as an EPDS score of 10 or higher before birth.

### 2.6 Postpartum follow-up

According to the 10th Revision of International and Statistical Classification of Diseases and Related Health Problems (ICD-10), the time point for PPD screening was set at 6 weeks after delivery in this study [2]. In addition, we wanted to investigate the association of psychological disorders in the early period after delivery and PPD. Thus, the EPDS scores were recorded both at 3 days and 6 weeks after delivery. PPD was defined as EPDS score ≥10 at 6 weeks after giving birth. Additionally, the pain at rest and in motion was assessed by NRS scores at 1, 2, and 3 days, and 6 weeks after giving birth. If the patient felt pain while lying in a supine position on the bed, it was defined as pain at rest; if the patient felt pain when coughing or walking on level ground, it was considered as pain in motion. Any adverse event was recorded within 3 days and 6 weeks after giving birth. Duration of labor and neonatal variables (neonatal gender, body weight, Apgar score 1, 5, and 10 min after giving birth) were followed. In this study, duration of labor was defined as the time from the beginning of regular contractions (lasting for more than 30 s with an interval of 5–6 min) to the end of giving birth for group LA and the operation time for group CS. Hospital stays were also recorded in this study. In China, mothers are traditionally expected to rest indoors for one full month after giving birth, so the data on companionship during this period (either by professional maid, parturient’s parents, or/and parents-in-law) and infant feeding choices (formula milk or/and breast milk) were also recorded.

### 2.7 Sample calculation and statistical analysis

According to the previous report, we assumed that the incidence of PPD would be 20% for subjects who underwent CS and 14% for parturients who received LA [22,25]. Hundred subjects in each group are required to obtain a statistical power of 80% at the significance level of 0.05, and the final sample size was 110 subjects considering a 10% dropout rate. The sample size was calculated by two independent proportions of power analyses of PASS 11.

Data were expressed as either mean value ± standard deviation (normal distribution data) or median (interquartile range) (non-normal distribution data) for continuous variables or total number (percent frequency) for categorical variables. The results of continuous variables were compared by either the *t*-test for normal distributed data or the Wilcoxon rank-sum test for non-normal distributed data. The chi-square test was used to analyze the results of categorical variables. Fisher’s exact test was used for categorical variables when the number of events was less than 5. To avoid false positives in multiple comparisons, *P* value was adjusted by Benjamini-Hochberg with R software (reversion 4.0.2, http://www.R-project.org) [26]. The predicted factors of PPD were assessed with multivariate logistic regression analysis. All the perinatal variables in this study, including maternal and neonatal factors, were included in the univariate analysis to screen for potentially predictive factors. To avoid missing some potential important variables, factors with a *P* value ≤0.1 in univariate analysis were defined as candidate variables [27]. Since a substantial bias of estimated regression coefficients may be generated via the stepwise method [28], all the potential variables were included in the multivariable model to determine the predictive factors by using the method of enter procedure. *P* value < 0.05 was considered as statistically significant.


**Informed consent:** Informed consent has been obtained from all the individuals included in this study.
**Ethical approval:** The research related to human use has been complied with all the relevant national regulations, institutional policies, and in accordance with the tenets of the Helsinki Declaration, and has been approved by the ethics committees of the First Affiliated Hospital of Chongqing Medical University (No. 2018-100). This study was registered in the Chinese Clinical Trial Registry (No. ChiCTR1900020510).

## 3 Results

**Figure 1:**
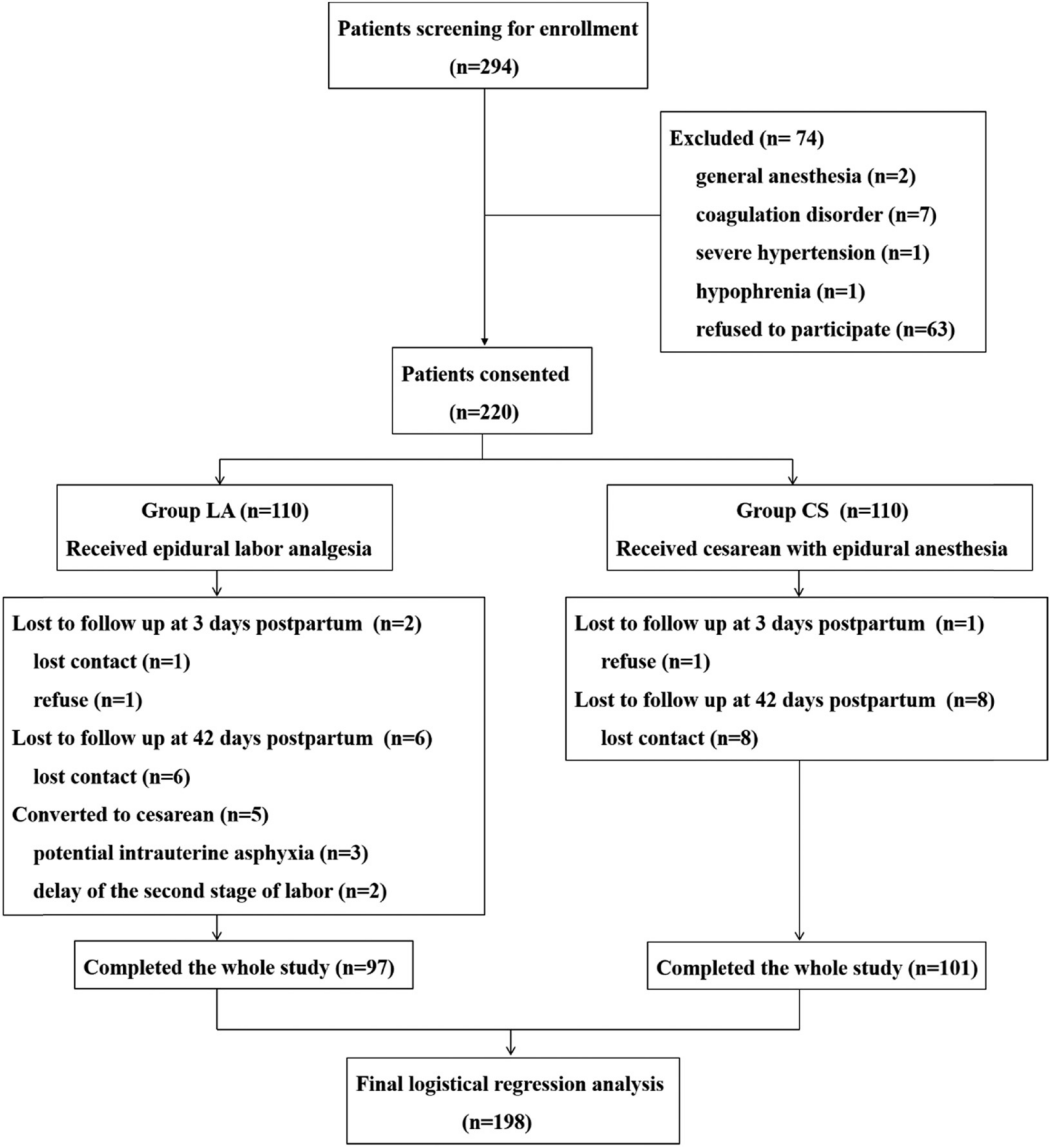
Flow chart of the study.

Among the 294 screened patients, 74 were excluded for not meeting the criteria or refusing to participate. The remaining 220 parturients were allocated to either group CS or group LA. Thirteen subjects in group LA and nine subjects in group CS did not complete the follow-up. Ultimately, 97 patients in group LA and 101 patients in group CS were included in the data analysis (Figure 1).

### 3.1 Baseline characteristics of eligible parturients

The baseline characteristics of the subjects are shown in Table 1. Parturients in group CS were older and had higher BMI compared with those in group LA (*P <* 0.001). Additionally, gravidity and parity in group CS were also higher compared to group LA (*P <* 0.001). On the other hand, more parturients in group LA attended the perinatal classes during pregnancy compared to group CS (*P <* 0.001). Besides, the baseline NRS scores were higher in group LA than group CS (*P <* 0.001). Other parameters were not significantly different between the two groups (*P* > 0.05).

**Table 1:** Baseline characteristics of eligible patients

Parameters	Group LA (*n* = 97)	Group CS (*n* = 101)	*P* value	Adjusted *P* value*
Age (years)	27.88 ± 3.34	31.69 ± 4.39	<0.001	0.001
BMI (kg/m^2^)	26.20 ± 2.66	27.34 ± 2.95	<0.001	0.001
Education >12 years	86 (88.65%)	83 (82.17%)	0.197	0.240
Family income (10,000 CNY/month)	0.83 ± 0.29	0.84 ± 0.26	0.790	0.790
Gravidity	1 (1–2)	3 (2–5)	<0.001	0.001
Parity	0 (0–0)	1 (0–1)	<0.001	0.001
Attending perinatal classes	41 (42.26%)	19 (18.81%)	<0.001	0.001
Present obstetrical and gynecological disease ^a^	49 (50.51%)	32 (31.68%)	0.015	0.022
Disease complicating pregnancy ^b^	44 (45.46%)	63 (62.37%)	0.016	0.022
Antenatal depression	21 (21.64%)	26 (25.74%)	0.499	0.548
Baseline NRS score	7.38 ± 1.26	2.32 ± 1.43	<0.001	0.001

BMI: body mass index and NRS: numerical rating scale.

Data were shown as mean value ± SD, median (range), or number of subjects (percentage).

^a^ Including premature rupture of membranes, potential placenta implantation, placenta previa, oligohydramnion, colpitis, hysteromyoma, and polycystic ovarian syndrome.

^b^ Including diabetes, hypertension, anemia, thyroid dysfunction, streptococcal infection, respiratory infection, cholestasis, systematic lupus erythematosus (SLE), and positive hepatitis B surface antigen.

*P value was adjusted using the method of Benjamini-Hochberg.

### 3.2 Difference of perinatal variables within 6 weeks follow-up

A significant difference was found in EPDS scores at 3 days after giving birth between the two groups, and group LA showed lower EPDS scores compared to group CS (*P* = 0.003). Similarly, EPDS scores at 6 weeks after delivery were also lower in group LA compared to group CS (*P* = 0.014). Notably, 11 in group LA and 26 patients in group CS were defined as PPD at 6 weeks after giving birth, and the incidence of PPD in group LA was lower compared to group CS (*P* = 0.011) (Table 2).

**Table 2:** Perinatal variables of postpartum follow-up

Parameters	Group LA (*n* = 97)	Group CS (*n* = 101)	*P* value	Adjusted *P* value*
NRS at rest after delivery				
1 day	2 (1–3)	2 (1–3)	0.598	0.721
2 days	1 (0–2)	1 (0–1.5)	0.058	0.092
3 days	0 (0–1)	0 (0–0.5)	0.001	0.005
6 weeks	0 (0–0)	0 (0–0)	0.869	0.869
NRS in motion after delivery				
1 day	3 (2–5)	4 (3–6)	0.002	0.008
2 days	2 (1–4)	2 (2–4)	0.743	0.792
3 days	1 (1–2.5)	1 (1–2)	0.631	0.721
6 weeks	0 (0–0)	0 (0–1)	<0.001	0.005
EPDS scores after delivery				
3 days	4 (1–7)	7 (3–9)	0.003	0.009
6 weeks	4 (1–7)	6 (2–10)	0.014	0.028
Adverse event after delivery ^a^				
3 days	29 (29.89%)	18 (17.82%)	0.046	0.081
6 weeks	22 (22.68%)	31 (30.69%)	0.203	0.270
Duration of labor (min)	691 (581–823)	40 (35–47)	<0.001	0.005
Hospital stay (days)	2.08 ± 0.27	4.2 ± 0.66	<0.001	0.005
Infant feeding			0.099	0.144
Formula milk	53 (54.63%)	49 (48.51%)		
Breast milk	1 (1.03%)	7 (6.93%)		
Mixed feeding	43 (44.34%)	45 (44.56%)		
Main companion			0.01	0.025
Professional maid	15 (15.46%)	25 (24.75%)		
Parents-in-law	23 (23.71%)	31 (30.69%)		
Parturient’s parents	19 (19.58%)	32 (31.68%)		
Mixed companion	40 (41.25%)	22 (12.88%)		
Occurrence of PPD	11 (11.34%)	26 (25.74)	0.011	0.025

NRS: numerical rating scale, EPDS: Edinburgh Postnatal Depression Scale, and PPD: postpartum depression.

Data were shown as number of subjects (percentage) or median (range).

^a^Adverse event included milk spitting up, jaundice, bacterial or viral infection, pneumonia, allergy, diarrhea, hospitalization of the baby, and bellyache, lumbago and back pain, vaginal bleeding, dizziness, numbness of legs, mastitis, fever, uroclepsia, hydrosis, perineal lacerations, placenta remnant, nausea and vomiting, constipation, chest congestion, and palpitation.

*P value was adjusted using the method of Benjamini-Hochberg.

The parturients in group CS spent much less time giving birth than their counterparts in group LA (*P <* 0.001). However, they tend to stay much longer in hospital (*P <* 0.001). Besides, NRS scores were higher in group CS at rest at 3 days *(P* = 0.001), in motion at 1 day (*P* = 0.002), and at 6 weeks *(P <* 0.001) after birth, but no significant difference was found at other time points within the 6 weeks follow-up (*P* > 0.05). Additionally, 29 cases in group CS and 18 cases in group LA experienced adverse events at 3 days after giving birth, and the incidence of adverse events was significantly lower in group CS compared to group LA (*P* = 0.046). Nevertheless, such significant difference disappeared after applying Benjamini-Hochberg correction (*P* = 0.081). No difference in the occurrence of adverse events at 6 weeks after giving birth were observed (*P* = 0.203), and no significant difference in feeding modalities between the two groups were found (*P* = 0.099). However, a significant difference was found in terms of the main companion during the 1-month indoor maternal rest (*P* = 0.01) (Table 2).

In addition, variables of neonates were also recorded. and no differences between the two groups were observed in terms of the gender, body weight, or Apgar scores at 1, 5, and 10 min after giving birth (*P* > 0.05) (Table 3).

**Table 3:** Neonatal variables of parturients

Parameters	Group LA (*n* = 97)	Group CS (*n* = 101)	*P* value	Adjusted *P* value*
Gender			0.462	0.610
Male	42 (43.29%)	49 (48.51%)		
Female	55 (56.71%)	52 (51.49%)		
Body weight (g)	3344.58 ± 312.34	3404.95 ± 404.19	0.242	0.610
Apgar score after birth				
1 min	10 (9–10)	10 (9–10)	0.610	0.610
5 min	10 (10–10)	10 (10–10)	0.534	0.610
10 min	10 (10–10)	10 (10–10)	0.308	0.610

Data were shown as mean value ± SD, median (range), or number of subjects (percentage).

*P value was adjusted using the method of Benjamini-Hochberg.

### 3.3 Factors associated with PPD

Thirty-one variables were included in the univariate analysis to screen for potentially predictive factors of PPD, and 8 variables were selected as candidate variables, including LA, family income, duration of labor, antenatal depression, attending perinatal classes, neonate of male, EPDS at 3 days after delivery, and adverse event at 6 weeks after delivery. All the candidate variables were included in the multivariate logistic regression model using the enter method, and three of them were identified as the independent predictors, namely LA (OR 0.049; 95% CI, 0.003–0.955; and *P* = 0.047), family income (OR 7.267; 95% CI, 1.65–32.014; and *P* = 0.009), and EPDS at 3 days after birth (OR 1.275; 95% CI, 1.14–1.426; and *P <* 0.001). Hosmer-Lemeshow test suggested the model to be a good fit (*χ* ^2^ = 9.758, df = 8, and *P* = 0.282) (Table 4).

**Table 4:** Univariate and multivariate regression analysis of PPD at 6 weeks after delivery

Parameters	Univariate		Multivariate	
	OR (95% CI)	*P*	OR (95% CI)	*P*
LA	0.369 (0.171–0.797)	0.011	0.049 (0.003–0.955)	0.047
Age	1.011 (0.932–1.097)	0.79		
Present obstetrical and gynecological disease	1.141 (0.547–2.38)	0.724		
Disease complicating pregnancy	0.874 (0.425–1.795)	0.713		
BMI	1.052 (0.931–1.19)	0.415		
Family income	5.365 (1.598–18.004)	0.007	7.267 (1.65–32.014)	0.009
NRS baseline	0.927 (0.817–1.051)	0.235		
Duration of labor	0.999 (0.998–1)	0.088		
Antenatal depression	5.014 (2.339–10.75)	<0.001		
Education	0.458 (0.131–1.604)	0.222		
Gravidity	1.225 (0.947–1.585)	0.122		
Parity	1.662 (0.843–3.278)	0.143		
Attending perinatal classes	2.103 (0.867–5.101)	0.1		
Hospital stay	1.253 (0.934–1.68)	0.132		
Neonate of male	1.907 (0.922–3.943)	0.082		
Neonatal body weight	1 (0.999–1.001)	0.406		
Apgar score 1 min after delivery	0.894 (0.576–1.387)	0.617		
Apgar score 5 min after delivery	0.78 (0.123–4.941)	0.792		
Apgar score 10 min after delivery		1		
NRS at rest 1 day after delivery	1.073 (0.836–1.376)	0.58		
NRS in motion 1 day after delivery	1.14 (0.915–1.422)	0.243		
NRS at rest 2 days after delivery	1.059 (0.784–1.432)	0.707		
NRS in motion 2 days after delivery	1.063 (0.817–1.383)	0.65		
NRS at rest 3 days after delivery	1.074 (0.675–1.71)	0.762		
NRS in motion 3 days after delivery	1.077 (0.775–1.495)	0.66		
Adverse event at 3 days after delivery	1.417 (0.578–3.474)	0.447		
EPDS at 3 days after delivery	1.281 (1.17–1.402)	<0.001	1.275 (1.14–1.426)	<0.001
NRS scores at rest 6 weeks after delivery	1.004 (0.294–3.43)	0.995		
NRS scores in motion 6 weeks after delivery	1.11 (0.729–1.629)	0.627		
Adverse event at 6 weeks after delivery	0.525 (0.247–1.118)	0.095		
Infant feeding				
Formula milk	1 (reference)			
Breast milk	1.456 (0.272–7.783)	0.66		
Mixed feeding	0.971 (0.465–2.027)	0.937		
Central companion				
Professional maid	1 (reference)			
Parents-in-law	1.2 (0.425–3.391)	0.731		
Parturient’s parents	0.735 (0.243–2.224)	0.585		
Mixed companion	0.436 (0.138–1.381)	0.158		

NRS: numerical rating scale, EPDS: Edinburgh Postnatal Depression Scale, OR: odds ratio, CI: confidence interval; Hosmer-Lemeshow test of the model: *χ* ^2^ = 9.758, df = 8, and *P* = 0.282.

## 4 Discussion

In the present study, we found that parturients who received LA exhibited a lower incidence of PPD compared to those who underwent CS. LA is identified as an independent factor associated with a decreased risk of PPD. On the other hand, high family income and EPDS scores 3 days after giving birth were associated with an increased risk of PPD.

With the advancement of our understanding of PPD, some risk factors have been identified. However, the role of delivery modes is still controversial. The large-scale studies evaluating effect of delivery modes (CS against spontaneous vaginal delivery) on PPD have reported completely opposite results [29,30]. Certainly, race, culture, and localization probably explain the difference in PPD incidence to some extent. However, to the best of our knowledge, two important factors, namely the intensity of labor pain and the parturient’s preference, should not be ignored when considering the difference between these two delivery modes. First of all, previous study suggested that labor pain is related to a mood disorder in the early postpartum period [19], and Lim et al. have confirmed that reliefs in labor pain are associated with a decreased risk of PPD [31]. Undoubtedly, the experience of labor pain is totally different between the CS and spontaneous vaginal delivery, which could possibly explain the varied risks of PPD. Second, it has been reported that women who have a strong antepartum preference for vaginal delivery but end up delivering by CS are at higher risks of PPD [32]. To clarify the effects of the two delivery modes on PPD one step further, women who experienced vaginal delivery with epidural analgesia or elective CS based on their own maternity preference were included in our study. The results showed that the incidence of PPD were lower in women who received LA than those who underwent CS, and LA was identified as an independent factor associated with a decreased risk of PPD. As far as we know, no previous reports have addressed the influence of LA and CS on PPD. Our study draws a precise conclusion about the effect of the two delivery modes on PPD. Besides, our study suggested that special attention should be paid to concomitant factors when considering the effects of delivery modes on PPD. Further studies are needed to clarify the influences of the various submodes of delivery (elective/emergency CS, spontaneous vaginal delivery, LA, vaginal delivery with forceps, etc.) on PPD.

Patients with low family income usually have to deal with more difficulties in life, and they tend to worry more about the future. Previous literature suggested that women with a low family income was associated with an increased risk of PPD [33,34]. However, some opposite results were also reported, Chaudron et al. found there was no relationship between family income and PPD [35]. Interestingly, in the present study, we found women with high family income were susceptible to PPD. A reasonable explanation is that even the relatively lower family income in our study was sufficient for the mothers to cover the daily cost and cope the diverse problems in life. According to the official statistics, the average personal income per month in Chongqing (China) is 4,639 CNY in 2019 [36]. In our study, the low family income defined was close to that amount, suggesting that even the women with a low family income could lead a comfortable life. Besides, more money does not mean worry-free, and patients with a high family income may not lead a carefree life as they may face more interpersonal, social, and business stress. A previous study reported that children living in wealthy families are more prone to suffer from serious depression and anxiety disorders [37]. However, the effect of family income on PPD still needs further verifications, and the difference between the definitions of low and high incomes should be considered, as well.

Characteristics of eligible patients were not comparable in this study, women who underwent CS were older and had higher BMI than those who received LA. However, age was not included as predictor for PPD in the final multivariate regression analysis. These results were inconsistent with previous findings, which suggested that the risk of PPD was higher for older mothers than younger ones [38]. Such a phenomenon can be explained because age seems to exhibit a segmental effect on PPD. All the positive results were found when comparing women ≥35 years with younger ones [38,39]. Unlike the previous designs, age was regarded as a continuous variable in our study, and most of the eligible women were younger than 35 years. Therefore, we found the risk of PPD was not positively correlated with age. Besides, BMI was also confirmed to be not associated with PPD in this study. However, previous study reported that maternal overweight and obesity during pregnancy were associated with PPD [40]. Recently, a large-scale study including about 700,000 women demonstrated that pregnancy BMI is related to PPD risk. Particularly, low BMI was associated with an increased risk of PPD, while overweight increased the risk only in women without a history of depression. In other words, the risk was modified by depression history [41]. In our opinion, the influence of BMI on PPD is complicated and uncertain, especially under potential confounding factors. Much more studies are needed not only to confirm the relationship between BMI and PPD but also to investigate the adjustment effect of BMI on PPD with some potential confounding factors.

EPDS is a self-report and user-friendly questionnaire that was used to evaluate PPD in the present study. However, it should be noted that the EPDS is just a screening scale for PPD used by non-psychiatrists, and it is not a professional’s tool for mental diagnosis. PPD has been assessed by EPDS in many studies, mainly because it has an estimated 80% sensitivity for diagnosis of depression [42]. Meanwhile, high PPD scores have been suggested as strong predictors of PPD [43,44]. In this study, we found that high EPDS score at 3 days postpartum was an independent risk factor of PPD. It can assist the doctors in the early identification of the patients with a high risk of PPD, prompting timely intervention to prevent the undesired condition. In the present study, the time point to assess PPD was chosen at 6 weeks postpartum as proposed by several previous studies [45,46,47]. Nonetheless, other time points have also been reported [48,49,50]. At present, there is no universally accepted time point for PPD assessment, and it still remains unclear whether different assessment time points could influence PPD results.

The present study has several limitations. First, patients make their own decision on the delivery modes in our study, so randomization and blinding could not be conducted in this study, it is not representative for all women who undergo CS or LA. Second, EPDS was used as the only evaluation scale to screen antenatal depression in this study, and psychiatrists were not involved in the study by providing any professional diagnosis. Patients were not excluded for the sole reason of positive EPDS scores, which may affect the final outcome. Third, personality trait is an important influential factor for the psychological condition of a person, and it might make a difference in PPD. However, it was not assessed in our study. Finally, the baseline characteristics were not compared between the two groups in this observational study; therefore, the causal relationship between the delivery modes and outcomes could only be identified. Further confirmations are needed to make our findings be of any practical value.

## 5 Conclusion

Compared to the CS, LA was more effective in reducing the incidence of PPD. LA was regarded as a protective factor of PPD; however, high family income and EPDS scores at 3 days postpartum were associated with an increased risk of PPD, and the clinical significance of LA in PPD prevention needs further verification.
